# Extradigital glomus tumor in the deltopectoral region^؆^

**DOI:** 10.1016/j.abd.2026.501407

**Published:** 2026-06-22

**Authors:** Mauricio Sandoval Osses, Karol Baksai Elespuru, Camila Tais Escobar Soto, Diego San Martín Morandé

**Affiliations:** a Department of Dermatology, Clínica Las Condes, Santiago, Chile; b Department of Pathological Anatomy, Clínica Las Condes, Santiago, Chile; c School of Medicine, Universidad Finis Terrae, Santiago, Chile

Dear Editor,

Glomus tumors are rare benign neoplasms originating from the glomus cells of the afferent arteriole of the glomus body, a structure involved in thermoregulation. Although they account for less than 2% of soft tissue tumors of the hand, their extradigital presentation is considerably rarer and may occur in the extremities, trunk, or head and neck.^1^, ^2^

The extradigital form, which often lacks the classic clinical pattern of intense localized pain exacerbated by cold, is frequently diagnosed late or misdiagnosed as other benign or malignant cutaneous or subcutaneous conditions.^3^

A clinical case is presented involving a 76-year-old man with no previous history of malignancy who presented with a lesion in the left deltopectoral region. Four years earlier, the patient had noticed an asymptomatic cutaneous lesion in the same area. At that time, the lesion was surgically excised, and according to the medical record, histopathological examination was consistent with an angioma; however, the original histopathological report was not available for review.

Approximately two years after the excision, a new lesion appeared at the site of the previous surgical scar. Initially, this recurrent lesion was asymptomatic and presented as a small papule. Six months prior to the current consultation, the patient reported the onset of progressive pain, described as sharp and constant, with a Visual Analog Scale (VAS) score of 6/10, without exacerbation upon exposure to cold.

Physical examination revealed a single raised papular lesion measuring approximately 5 mm, located centrally within an overlying 2 cm whitish linear scar and surrounded by violaceous discoloration, without signs of acute inflammation (Fig. 1). The lesion was painful on palpation, mobile, and not adherent to deeper planes. No palpable lymphadenopathy or other associated cutaneous lesions were identified.

**Fig. 1:**
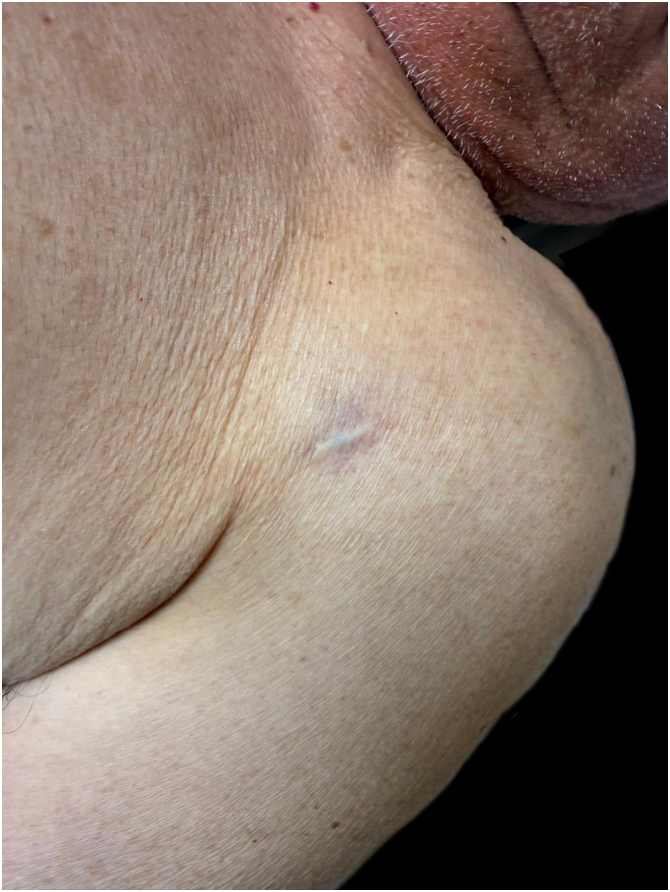
Clinical photograph of a solitary deltopectoral papular lesion (∼5 mm), located centrally within a whitish linear scar (∼2 cm), surrounded by violaceous discoloration.

Complete excision of the lesion was performed. Histopathological examination revealed proliferation of uniform, rounded cells with eosinophilic cytoplasm arranged around thin-walled vessels, consistent with a benign solid glomus tumor (Fig. 2). Immunohistochemical examination showed negative staining for CD34 in lesional cells and positive staining for smooth muscle actin (Fig. 3).

**Fig. 2:**
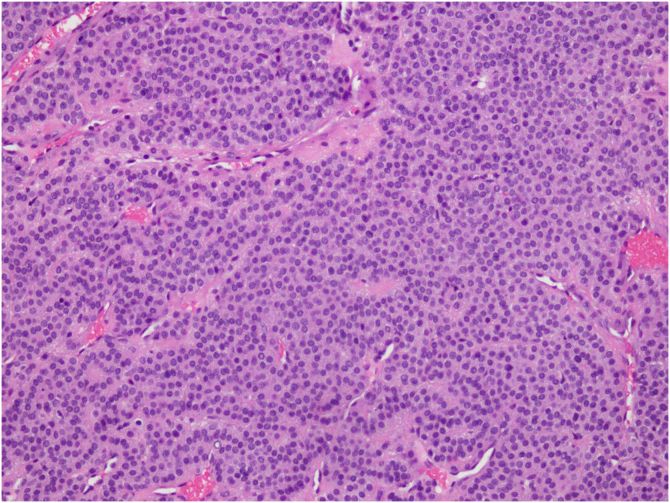
Hematoxylin & eosin (400×). Proliferation of monomorphic glomus cells with round nuclei and eosinophilic cytoplasm, arranged in nests and sheets around thin-walled blood vessels.

**Fig. 3:**
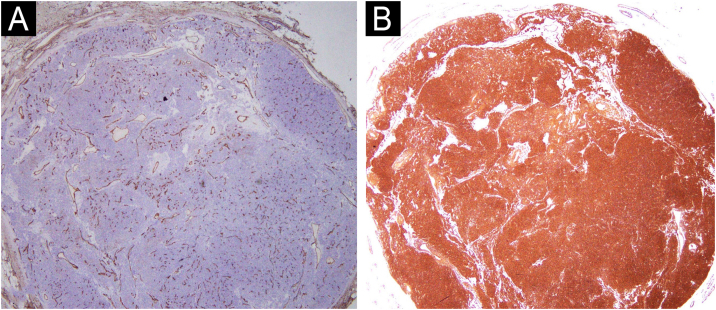
Immunohistochemistry. (A) CD34, negative staining in lesional cells (20×). (B) Positive smooth muscle actin in lesional cells (20×).

Extradigital glomus tumors constitute a minority of cases, with an estimated incidence of less than 10%.^4^ Their clinical presentation is heterogeneous, contributing to diagnostic delays that, according to recent series, can exceed five years from symptom onset.^2^ Although the exact pathogenesis remains unknown, trauma has been proposed as a potential etiological factor in the development of glomus tumors.^5^

The differential diagnoses include epidermoid cysts, dermatofibromas, cutaneous leiomyomas, and cutaneous metastases, among others. High-resolution ultrasound and Magnetic Resonance Imaging (MRI) are useful for characterization. Ultrasound typically demonstrates a well-defined, solid, hypoechoic lesion, while MRI reveals hypointense signal intensity on T1-weighted images and hyperintense signal intensity on T2-weighted images.^6^

Complete surgical excision is the treatment of choice and is curative in most cases, providing immediate pain relief. Recurrence is uncommon but may occur in cases of incomplete resection or multiple tumors.^1^, ^3^

In this case, the deltopectoral location and absence of cold sensitivity hindered initial clinical suspicion, consistent with previous reports in which extradigital presentation has led to misdiagnosis.^2^ Furthermore, a previous surgical intervention for a presumed angioma may have acted as a traumatic stimulus or, more likely, represented an incomplete excision of a pre-existing, misdiagnosed glomus tumor.

This case underscores the importance of considering extradigital glomus tumor in the differential diagnosis of painful papular or nodular lesions, even in atypical locations such as the trunk. Clinical recognition supported by imaging facilitates precise surgical planning, and histopathological examination remains the definitive diagnostic tool.

ORCID ID

Mauricio Sandoval Osses: 0000-0003-2234-6187

Karol Baksai Elespuru: 0009-0007-6065-1218

Camila Tais Escobar Soto: 0009-0004-2852-898X

Diego San Martín Morandé: 0009-0003-0191-8657

## Financial support

This research did not receive any specific grant from funding agencies in the public, commercial, or not-for-profit sectors.

## Authors' contributions

Mauricio Sandoval Osses: Conceptualization; methodology; data curation; clinical management/decision-making; final approval.

Karol Baksai Elespuru: Formal analysis; supervision.

Camila Tais Escobar Soto: Writing-original draft; data curation; formal analysis; literature review; supervision.

Diego San Martín Morandé: Supervision; literature review.

## Research data availability

Does not apply.

## Conflicts of interest

None declared.

## References

[1] Del Carpio G.S., Burgos E.M.P., Pozo Kreilinger J.J., Bernabeu Taboada D., Peletero Pensado M., Tapia Viñé M. (2024). Case series of extradigital glomus tumors: imaging findings, differential diagnosis and radiologic-pathologic correlation. Egypt J Radiol Nucl Med..

[2] Cohen P.R. (2023). Glomus extradigital tumor: a case report of an extradigital glomus tumor on the wrist and comprehensive review of glomus tumors. Cureus..

[3] Gómez-Sánchez M.E., Alfageme-Roldán F., Roustán-Guillón G., Segurado-Rodriguez M.A. (2014). Tumores glómicos digitales y extradigitales: utilidad de la ecografía. Actas Dermosifiliogr..

[4] Nazir M. (2025). Diagnostic and surgical challenges in extradigital glomus tumour: a case report. EMJ Innov..

[5] Soufi G.J., Golabchi M.R., Sadeghizade S. (2024). Upper arm glomus tumor. Radiol Case Rep..

[6] Patel T., Meena V., Meena P. (2022). Hand and foot Glomus Tumors: significance of mri diagnosis followed by histopathological assessment. Cureus..

